# Predictors of treatment switching in the Big Multiple Sclerosis Data Network

**DOI:** 10.3389/fneur.2023.1274194

**Published:** 2023-12-22

**Authors:** Tim Spelman, Melinda Magyari, Helmut Butzkueven, Anneke Van Der Walt, Sandra Vukusic, Maria Trojano, Pietro Iaffaldano, Dana Horáková, Jirí Drahota, Fabio Pellegrini, Robert Hyde, Pierre Duquette, Jeannette Lechner-Scott, Seyed Aidin Sajedi, Patrice Lalive, Vahid Shaygannejad, Serkan Ozakbas, Sara Eichau, Raed Alroughani, Murat Terzi, Marc Girard, Tomas Kalincik, Francois Grand'Maison, Olga Skibina, Samia J. Khoury, Bassem Yamout, Maria Jose Sa, Oliver Gerlach, Yolanda Blanco, Rana Karabudak, Celia Oreja-Guevara, Ayse Altintas, Stella Hughes, Pamela McCombe, Radek Ampapa, Koen de Gans, Chris McGuigan, Aysun Soysal, Julie Prevost, Nevin John, Jihad Inshasi, Leszek Stawiarz, Ali Manouchehrinia, Lars Forsberg, Finn Sellebjerg, Anna Glaser, Luigi Pontieri, Hanna Joensen, Peter Vestergaard Rasmussen, Tobias Sejbaek, Mai Bang Poulsen, Jeppe Romme Christensen, Matthias Kant, Morten Stilund, Henrik Mathiesen, Jan Hillert, The Big MS Data Network: a collaboration of the Czech MS Registry, the Danish MS Registry, Italian MS Registry, Swedish MS Registry, MSBase Study Group, and OFSEP

**Affiliations:** ^1^Department of Clinical Neuroscience, Karolinska Institute, Stockholm, Sweden; ^2^MSBase Foundation, Melbourne, VIC, Australia; ^3^The Danish Multiple Sclerosis Registry, Copenhagen University Hospital, Rigshospitalet, Glostrup, Denmark; ^4^Danish Multiple Sclerosis Center, Department of Neurology, Copenhagen University Hospital Rigshospitalet, Glostrup, Copenhagen, Denmark; ^5^MS and Neuroimmunology Research, Central Clinical School, Alfred and Box Hill Hospitals, Monash University, Melbourne, VIC, Australia; ^6^Department of Neuroscience, Central Clinical School, Monash University, Melbourne, VIC, Australia; ^7^Service de Neurologie, Sclérose en Plaques, Pathologies de la Myéline et Neuro-Inflammation, Hôpital Neurologique Pierre Wertheimer, Hospices Civils de Lyon, Lyon, France; ^8^Centre des Neurosciences de Lyon, L'Institut national de la santé et de la recherche médicale 1028 et Centre national de la recherche scientifique joint research units5292, Lyon, France; ^9^Faculté de Médicine Lyon-Est, Université Claude Bernard Lyon 1, Villeurbanne, Auvergne-Rhône-Alpes, France; ^10^Department of Basic Medical Sciences, Neurosciences and Sense Organs, University of Bari Aldo Moro, Bari, Italy; ^11^Department of Translational Biomedicine and Neuroscience, DiBraiN, University of Bari Aldo Moro, Bari, Italy; ^12^Department of Neurology and Center of Clinical Neuroscience, First Faculty of Medicine, Charles University and General University Hospital, Prague, Czechia; ^13^Biogen International GmbH, Zug, Switzerland; ^14^Biogen Digital Health, Biogen Spain, Madrid, Spain; ^15^University of Montreal Hospital Research Centre and Universite de Montreal, Montreal, QC, Canada; ^16^University Newcastle, Callaghan, NSW, Australia; ^17^Hunter Medical Research Institute, Hunter New England Health, John Hunter Hospital, New Lambton Heights, NSW, Australia; ^18^Department of Neurology, Neuroscience Research Center, Golestan University of Medical Sciences, Gogan, Iran; ^19^Faculty of Medicine, Division of Neurology, Geneva University Hospital, Geneva, Switzerland; ^20^Isfahan University of Medical Sciences, Isfahan, Iran; ^21^Dokuz Eylul University, Konak/Izmir, Türkiye; ^22^Department of Neurology, Hospital Universitario Virgen Macarena, Sevilla, Spain; ^23^Division of Neurology, Department of Medicine, Amiri Hospital, Sharq, Kuwait; ^24^Medical Faculty, 19 Mayis University, Samsun, Türkiye; ^25^Clinical Outcomes Research Unit, Department of Medicine, University of Melbourne, Melbourne, VIC, Australia; ^26^Neuro Rive-Sud, Longueuil, QC, Canada; ^27^Nehme and Therese Tohme Multiple Sclerosis Center, American University of Beirut Medical Center, Beirut, Lebanon; ^28^Department of Neurology, Faculty of Health Sciences, University Fernando Pessoa, Porto, Portugal; ^29^Academic MS Center Zuyderland, Department of Neurology, Zuyderland Medical Center, Sittard-Geleen, Netherlands; ^30^Center of Neuroimmunology, Service of Neurology, Hospital Clinic de Barcelona, Barcelona, Spain; ^31^Hacettepe University, Ankara, Türkiye; ^32^Department of Neurology, Hospital Clinico San Carlos, Madrid, Spain; ^33^Department of Neurology, School of Medicine and Koc University Research Center for Translational Medicine (KUTTAM), Koc University, Istanbul, Türkiye; ^34^Royal Victoria Hospital, Belfast, United Kingdom; ^35^University of Queensland, Brisbane, QLD, Australia; ^36^Nemocnice Jihlava, Jihlava, Czechia; ^37^Groene Hart Ziekenhuis, Gouda, Netherlands; ^38^St Vincent's University Hospital, Dublin, Ireland; ^39^Bakirkoy Education and Research Hospital for Psychiatric and Neurological Diseases, Istanbul, Türkiye; ^40^CSSS Saint-Jérôme, Saint-Jerome, QC, Canada; ^41^Monash Health, Melbourne, VIC, Australia; ^42^Rashid Hospital, Dubai, United Arab Emirates; ^43^Department of Neurology, Aarhus University Hospital, Aarhus, Denmark; ^44^Department of Neurology, Southwest Jutland Hospital, University Hospital of Southern Denmark, Esbjerg, Denmark; ^45^Department of Neurology, Nordsjællands Hospital, Hillerød, Denmark; ^46^Department of Neurology, Hospital of Southern Jutland, University of Southern Denmark, Aabenraa, Denmark; ^47^Department of Neurology, Physiotherapy and Occupational Therapy, Gødstrup Hospital, Herning, Denmark; ^48^NIDO | Centre for Research and Education, Gødstrup Hospital, Herning, Denmark; ^49^Department of Neurology, Copenhagen University Hospital Herlev and Gentofte, København, Denmark

**Keywords:** multiple sclerosis, disease modifying treatment (DMT), treatment switching, disease registry, real world evidence (RWE)

## Abstract

**Background:**

Treatment switching is a common challenge and opportunity in real-world clinical practice. Increasing diversity in disease-modifying treatments (DMTs) has generated interest in the identification of reliable and robust predictors of treatment switching across different countries, DMTs, and time periods.

**Objective:**

The objective of this retrospective, observational study was to identify independent predictors of treatment switching in a population of relapsing-remitting MS (RRMS) patients in the Big Multiple Sclerosis Data Network of national clinical registries, including the Italian MS registry, the OFSEP of France, the Danish MS registry, the Swedish national MS registry, and the international MSBase Registry.

**Methods:**

In this cohort study, we merged information on 269,822 treatment episodes in 110,326 patients from 1997 to 2018 from five clinical registries. Patients were included in the final pooled analysis set if they had initiated at least one DMT during the relapsing-remitting MS (RRMS) stage. Patients not diagnosed with RRMS or RRMS patients not initiating DMT therapy during the RRMS phase were excluded from the analysis. The primary study outcome was treatment switching. A multilevel mixed-effects shared frailty time-to-event model was used to identify independent predictors of treatment switching. The contributing MS registry was included in the pooled analysis as a random effect.

**Results:**

Every one-point increase in the Expanded Disability Status Scale (EDSS) score at treatment start was associated with 1.08 times the rate of subsequent switching, adjusting for age, sex, and calendar year (adjusted hazard ratio [aHR] 1.08; 95% CI 1.07–1.08). Women were associated with 1.11 times the rate of switching relative to men (95% CI 1.08–1.14), whilst older age was also associated with an increased rate of treatment switching. DMTs started between 2007 and 2012 were associated with 2.48 times the rate of switching relative to DMTs that began between 1996 and 2006 (aHR 2.48; 95% CI 2.48–2.56). DMTs started from 2013 onwards were more likely to switch relative to the earlier treatment epoch (aHR 8.09; 95% CI 7.79–8.41; reference = 1996–2006).

**Conclusion:**

Switching between DMTs is associated with female sex, age, and disability at baseline and has increased in frequency considerably in recent years as more treatment options have become available. Consideration of a patient's individual risk and tolerance profile needs to be taken into account when selecting the most appropriate switch therapy from an expanding array of treatment choices.

## Introduction

Multiple sclerosis (MS) is a chronic, autoimmune, and demyelinating neurological disorder. Accurate diagnosis, disease-modifying therapies, and symptom management are key components of managing MS to improve patients' quality of life and limit disability progression (1, 2). Treatment discontinuation and switching in multiple sclerosis (MS) is a common challenge and opportunity in real-world clinical practice. The increasingly diverse range of disease-modifying treatments (DMTs) currently available to patients and clinicians, coupled with frequent changes in reimbursement and treatment recommendations, has generated interest in the identification of reliable and robust predictors of treatment switching across different countries, DMTs, and epoch changes (3–9). Whilst effectiveness and tolerance remain key drivers of treatment failure, the precise role of demographic and clinical factors remains less clear (10, 11).

This study group previously reported that both rates and reasons for treatment interruption, encompassing both treatment switching and discontinuation, remained largely stable over a 20-year observation period, suggesting that treatment switching was primarily driven by the properties of DMTs themselves and less related to either risk management or market competition (12).

The identification of independent predictors of treatment switching and response in long-term, heterogeneous disease is likely to form a key component in the development of personalized prediction and healthcare in multiple sclerosis. The accuracy and robustness of personalized prediction in RRMS is a key challenge in improving generalisability, often requiring large and representative samples (13–15). A recent systematic review further reported a lack of validated predictive tools for the early and reliable identification of key drivers of treatment failure, such as disease progression (16).

The study aimed to identify independent predictors of treatment switching in the Big MS Data Network in a population of relapsing-remitting MS (RRMS) patients initiating DMT at least once over the course of their disease (12, 17).

## Materials and methods

### Study design and data

This study was a retrospective analysis of observational, real-world data sourced from five clinical MS registries included in the BMSD network project: the Italian MS registry, the OFSEP of France, the Danish MS registry, the Swedish national MS registry, and the international MSBase Registry. These registries have previously been described in detail (12, 18–22). Treatment episodes and associated patient data complying with minimum dataset requirements were individually extracted from the five contributing datasets and then pooled into a single combined dataset.

### Data harmonization and quality

Data quality checks were conducted before merging to minimize outcome assessment and follow-up bias in the cohorts under study. This included ensuring all variables required for the minimum analysis dataset had been extracted and transferred in the correct format, consistent across all five registries. The data were checked for duplicates and date inconsistencies covering key demographics, disease characteristics, and treatment dates. Data counts were then performed to assess the completeness of crucial variables. The harmonization and quality assurance processes have been previously described (12, 17).

### Inclusions and exclusions

Patients were included in the final pooled analysis set if they had initiated at least one DMT during the relapsing-remitting MS (RRMS) stage. Patients not diagnosed with RRMS or RRMS patients not initiating DMT therapy during the RRMS phase were excluded from the analysis.

Poser or McDonald criteria were used to confirm MS diagnosis, depending on the time the diagnosis was made in each registry. Subjects were defined as exposed to a DMT if they had received at least one injection/infusion (or at least a one-time consumption of an oral drug). The pre-study period preceding the index date, during which patients were required to have continuous medical service coverage, was defined as the time since the first recorded visit in each registry up until the baseline date. This pre-study period ensured a standard run-in period prior to DMT exposure and a standard period during which the diagnosis of MS was identified.

### Variables and definitions

DMT starts recorded between 1996 and 2018, inclusive, were included in the analysis. The primary outcome variable in this analysis was treatment switching. Treatment switching was defined as the initiation of a new DMT within 6 months of ceasing a preceding DMT, consistent with previous studies using data from these registries (3, 23). Demographic and clinical characteristics at treatment start analyzed for association with subsequent treatment switching included age, sex, disease duration, time since diagnosis, EDSS, and calendar year of treatment start. The calendar year was further categorized into three treatment epochs, capturing the platform interferon-β (IFNβ) and glatiramer acetate (GLA) only era (1996–2006), the introduction of natalizumab (NTZ) infusion (2007–2012), and the era of oral treatments (including fingolimod [FTY], dimethyl fumarate [DMF], and teriflunomide [TERI]) (2013–2018).

### Ethics

The studies involving human participants were reviewed and approved by each contributing registry to the Big MS Data Network according to their own ethics and operating and inclusion rules. Each registry is required to obtain its own approval prior to the provision of the data and pooling.

### Statistical analyses

Categorical variables were summarized using frequency and percentage. Continuous variables were summarized using mean and standard deviation (SD) or median and interquartile range (IQR) as appropriate. The statistical unit of the modeling analysis was the treatment episode. Patients were permitted to contribute multiple DMT episodes to the analysis. Associations between potential clinical and demographic factors at treatment start and the switching end-point were analyzed using a multilevel shared frailty survival model (24). This involved using a mixed-effects Cox model where an indicator variable representing each registry providing data to the pooled analysis was used as a random effect. Hazard proportionality was assessed via the analysis of scaled Schoenfeld residuals. All multivariate models were further assessed for collinearity and heteroskedasticity. The analysis was performed both across the entire treatment cohort and then stratified for treatment epoch (1996–2006, 2007–2012, and 2013 onwards) and drug/drug class (IFNβ, GLA, NTZ, FTY, DMF, and TERI) for which the sample size was sufficient, to assess whether the pattern of switching predictors varied by time and/or DMT product. All analyses were conducted on a complete-case basis, with no imputation for missing data. All analyses were performed in Stata version 16.1 (StataCorp, College Station, Texas) and R version 3.6.3 (R Foundation for Statistical Computing, Vienna, Austria).

## Results

### Patients and treatment episodes

A total pooled sample of 110,326 patients contributing 269,822 DMT treatment episodes from the five registries was included in the analysis (Table 1). A total of 78,629 (70.9%) of these patients were female. The mean (SD) age at disease onset across the pooled sample was 30.9 years (10.3), whilst the mean (SD) age at first treatment initiation was 36.6 years (11.0). Of the 184,013 observed DMT stops, 159,309 (86.6%) switched to an alternate DMT within 6 months. Alternate platform DMTs (IFNβ or GLA), NTZ, or FTY were the most frequently switched to drugs across the observation period.

**Table 1:** Baseline characteristics of patients by registry.

	**Category**	**Denmark**	**Sweden**	**OFSEP**	**Italy**	**MSBase**	**Total**
**Patient characteristics** ^*^							
Patient count - *n*		7,990	15,983	24,616	26,985	34,752	110,326
Sex - *n* (%)	Female	5,485 (68.7)	11,245 (70.4)	18,333 (74.5)	18,315 (67.9)	24,891 (71.6)	78,269 (70.9)
	Male	2,505 (31.4)	4,738 (29.6)	6,283 (25.5)	8,670 (32.1)	9,861 (28.4)	32,057 (29.1)
Age at MS onset (years) - mean (SD)		32.8 (9.9)	34.4 (12.8)	31.1 (9.5)	29.6 (9.7)	30.5 (9.9)	30.9 (10.3)
Age at first DMT (years) - mean (SD)		38.3 (12.8)	40.7 (12.4)	36.3 (10.3)	35.8 (10.7)	35.5 (10.7)	36.6 (11.0)
**Treatment characteristics**							
Treatment episodes - *n*		14,252	38,229	65,535	79,816	71,990	269,822
Discontinuations – *n* (%)		8,936 (62.7)	24,704 (64.6)	45,966 (70.1)	59,590 (74.7)	44,817 (62.3)	184,013 (68.2)
Treatment duration (years) – mean (SD)		2.82 (2.38)	2.31 (2.24)	2.18 (2.18)	2.06 (2.12)	2.29 (2.20)	2.23 (2.20)

^*^Count of individual patients contributing at least 1 treatment episode to the analysis.

### Associations between baseline factors and treatment switching

Across the entire pooled sample of treatments, every one-point increase in EDSS at the start of treatment was associated with 1.08 times the rate of subsequent switching, adjusting for age, sex, and calendar year (adjusted hazard ratio [aHR] 1.08; 95% CI 1.07–1.08) (Table 2). Female sex was associated with 1.11 times the rate of switching relative to male sex (HR 1.11; 95% CI 1.08–1.14), whilst older age at baseline was also associated with an increased rate of treatment switching. DMTs started between 2007 and 2012 were associated with 2.48 times the odds of treatment switching relative to DMTs started between 1996 and 2006 (aHR 2.48; 95% CI 2.48–2.56), controlling for age, sex, and baseline EDSS. DMTs commenced from 2013 onwards were even more likely to switch relative to the earlier treatment epoch (aHR 8.09; 95% CI 7.79–8.41; reference = 1996–2006).

**Table 2:** Associations between baseline factors and treatment switching (shared frailty survival model).

**Factor at treatment start**	**Level**	**Unadjusted HR (95% CI) *p*-value**	**Adjusted HR (95% CI) *p*-value**
Gender	Females	1.09 (1.06, 1.11) <0.001	1.11 (1.08, 1.14) <0.001
	Males	Reference	Reference
EDSS at treatment start		1.07 (1.06, 1.07) <0.001	1.08 (1.07, 1.08) <0.001
Age at treatment start (10 year units)		1.16 (1.15, 1.17) <0.001	1.04 (1.03, 1.05) <0.001
Disease duration at treatment start^a^		1.03 (1.03, 1.03) <0.001	
Years since diagnosis^a^		1.04 (1.04, 1.05) <0.001	
Calendar year of treatment start^b^		1.16 (1.15, 1.16) <0.001	
Treatment epoch	1996–2006	Reference	Reference
	2007–2012	2.01 (1.97, 2.05) <0.001	2.48 (2.40, 2.56) <0.001
	2013+	5.67 (5.54, 5.81) <0.001	8.09 (7.79, 8.41) <0.001

^a^Collinear with age at treatment start: ^b^collinear with treatment epoch.

### Switching by epoch

Similar patterns of association were observed when the multilevel modeling was stratified by treatment epoch. When the analysis was limited to the earlier platform DMTs epoch (1996–2006), EDSS at treatment initiation demonstrated a larger influence on subsequent switching relative to the full observation period; every unit of EDSS higher at treatment start was associated with 1.14 times the rate of treatment switching (aHR 1.14; 95% CI 1.13–1.16) (Table 3). Women were associated with 1.13 times the rate of switching compared to men (aHR 1.13; 95% CI 1.07–1.20), whilst DMTs started in later calendar years within the 1996–2006 epoch were associated with greater odds of treatment switching (calendar year aHR 1.17; 95% CI 1.16, 1.18).

**Table 3:** Associations between baseline factors and treatment switching–stratified by treatment epoch.

**Factor at treatment start**	**Treatment epoch**		
	**1996–2006**	**2007–2012**	**2013**+
	**Adjusted HR (95% CI)** ***p*****-value**	**Adjusted HR (95% CI)** ***p*****-value**	**Adjusted HR (95% CI)** ***p*****-value**
Age at treatment start (10 year units)	1.05 (1.03, 1.08) <0.001	1.05 (1.04, 1.06) <0.001	1.14 (1.11, 1.16) <0.001
Female sex	1.13 (1.07, 1.20) <0.001	1.11 (1.07, 1.16) <0.001	1.11 (1.06, 1.17) <0.001
EDSS	1.14 (1.13, 1.16) <0.001	1.10 (1.09, 1.11) <0.001	1.02 (1.01, 1.03) 0.001
Calendar year	1.17 (1.16, 1.18) <0.001	1.24 (1.22, 1.25) <0.001	1.08 (1.06, 1.10) <0.001

Associations were again similar when the analysis was confined to the 2007–2013 epoch (Table 4). Higher EDSS at treatment start (aHR 1.10; 95% CI 1.09–1.11), female sex (aHR 1.11; 95% CI 1.07–1.16), older age at treatment start (aHR 1.01; 95% CI 1.00–1.01), and later calendar years (aHR 1.24; 95% CI 1.22–1.25) were all associated with a significantly increased rate of treatment switching. When the modeling was limited to the most recent treatment epoch (2013 onwards), baseline EDSS was associated with a significant, albeit considerably smaller, increase in switching rate (relative to the 1996–2006 and 2007–2012 treatment epochs), with every one-point increase in EDSS being associated with 1.02 times the rate of switching on adjusted modeling (aHR 1.02; 95% CI 1.01–1.03). Older age at treatment start, female sex, and later calendar years were all associated with significantly increased switching rates within the 2013+ epoch.

**Table 4:** Associations between baseline factors and treatment switching–stratified by DMT.

**Factor at treatment start**	**DMT**					
	**IFN**β	**Glatiramer Acetate**	**Natalizumab**	**Fingolimod**	**Dimethyl fumarate**	**Teriflunomide**
	**Adjusted HR (95% CI)** ***p*****-value**	**Adjusted HR (95% CI)** ***p-*****value**	**Adjusted HR (95% CI)** ***p*****-value**	**Adjusted HR (95% CI)** ***p*****-value**	**Adjusted HR (95% CI)** ***p*****-value**	**Adjusted HR (95% CI)** ***p*****-value**
Age at treatment start (10 year units)	1.05 (1.02, 1.07) <0.001	0.96 (0.93, 0.99) 0.019	1.02 (0.99, 1.05) 0.061	1.02 (0.99, 1.06) 0.166	1.08 (1.02, 1.13) 0.003	1.21 (1.13, 1.30) <0.001
Female sex	1.17 (1.11, 1.24) <0.001	1.03 (0.96, 1.11) 0.351	1.03 (0.97, 1.11) 0.335	1.05 (0.97, 1.14) 0.212	1.11 (0.98, 1.25) 0.102	1.26 (1.04, 1.51) 0.015
EDSS	1.09 (1.08, 1.11) <0.001	1.12 (1.10, 1.14) <0.001	1.01 (1.00, 1.03) 0.079	0.99 (0.99, 1.01) 0.273	0.99 (0.95, 1.01) 0.130	0.98 (0.93, 1.02) 0.293
Calendar year	1.15 (1.14, 1.15) <0.001	1.14 (1.13, 1.15) <0.001	1.35 (1.33, 1.37) <0.001	1.06 (1.04, 1.08) <0.001	1.00 (0.94, 1.05) 0.878	1.03 (0.98, 1.09) 0.309

### Switching by DMT

Across the entire 1996–2018 observation period, higher EDSS at treatment start (aHR 1.09; 95% CI 1.08–1.11), female sex (aHR 1.17; 95% CI 1.11–1.24), older age (aHR 1.01; 95% CI 1.00–1.01), and calendar year (aHR 1.15; 95% CI 1.14, 1.15) were all associated with increased rates of switching from IFNβ (Table 4). A similar pattern of association was observed when the analysis was limited to GLA, although female sex was no longer associated with an increased rate of switching. Calendar year was again a major driver of switching from NTZ (aHR 1.35; 95% CI 1.33–1.37) and FTY (aHR 1.06; 95% CI 1.04–1.08), although no such association was observed with either DMF or TERI. Older age at baseline was associated with an increased rate of switching from both DMF and TERI. Women treated with TERI were also associated with 1.26 times the rate of switching compared to men treated with TERI (aHR 1.26; 95% CI 1.04–1.51).

## Discussion

As previously reported by this study group, treatment switching in RRMS is common. In this new analysis of the same cohort, we observed that older age, female sex, and higher EDSS at the time of index treatment initiation were consistently associated with a significantly higher switching rate. This is consistent with previous observations from registry studies, which have also reported sex and EDSS as independent predictors of treatment interruption in both RRMS and CIS (25). Kalincik et al.'s (26) multivariate predictive algorithm modeling of individual treatment response and persistence using a large number of demographic and clinical factors identified older age as an independent predictor of on-treatment relapse triggering discontinuation. Similarly, Ayrignac et al. (27) reported that patients with a baseline EDSS of 2 or more were strongly associated with treatment failure. A recent Danish study of 3,297 MS patients reported age and sex as key determinants of both the initial first-line DMT choice and subsequent escalation product (28). The most prominent effect, in terms of adjusted hazard ratio size, observed across the whole cohort was the treatment epoch, with later epochs (2007–2012 and 2013+) associated with progressively larger rates of treatment switching, relative to the earlier 1996–2006 epoch where lower-to-moderate efficacious platform DMTs dominated MS treatment and diagnostic delay was more common (29). This progressively larger rate of treatment switching with time likely reflects the relatively broader range of both DMT products available with varying effectiveness and safety profiles and an increase in the diversity of treatment strategies practiced during more recent years (30–32). It may also, in part, reflect a shift in treatment strategy toward the earlier introduction of high-efficacy treatments in response to treatment failure (3, 33).

Whilst the strongest predictors of treatment switching, such as age, sex, and baseline EDSS, were largely consistent across treatment epochs, there were some key differences in the patterns of predictors when the analysis was stratified by DMT. Higher baseline EDSS was a strong correlate of subsequent treatment switching in the older platform drugs (IFNβ and GLA), potentially secondary to longer disease duration whilst awaiting newer switch products to become available, but less so for NTZ (HR 1.01; 95% CI 1.00, 1.03). By further contrast, no association between EDSS and treatment switching was observed for more recent oral and/or higher efficacy preparations, including DMF, TERI, and FTY. This may in part be a function of the original platform DMDs being used more frequently across a broader range of EDSS scores compared with oral first-line therapies such as DMF or TERI. A sex effect was observed for IFNβ (female sex HR 1.17; 95% CI 1.11, 1.24) and TERI (HR 1.26; 95% CI 1.04, 1.51), but not for any of the other DMTs studied. The latter observation may be partially explained by the early TERI discontinuation associated with pregnancy planning. With the exception of TERI, treatment switching became progressively more common across most years in the later years of the observation period.

Consistent with the observation that the observed effect of EDSS at the time of treatment start on switching rates was maximal under the older platform DMTs was the additional observation that this baseline EDSS effect was largest during both the earlier 1996–2006 (HR 1.14; 95% CI 1.13, 1.16) and 2007–2012 periods (HR 1.10; 95% CI 1.09, 1.11). Whilst significant, the EDSS effect in the 2013 onwards period was comparatively much smaller (HR 1.02; 95% CI 1.01, 1.03). This may in part be due to the increasingly greater role of MRI and relapse activity in guiding treatment decisions (34, 35).

Our observation that treatment switching has become more common over time likely reflects, at least in part, a desire by clinicians and patients to optimize disease management, particularly in terms of delaying or preventing disability progression and maintaining neurological function (31, 36, 37). Identifying independent and reliable demographic and clinical predictors also has real-world implications for personalized medicine. A recent French cohort study used similar predictive factors to develop a dynamic scoring system to improve the timing of treatment switch decisions through the earlier identification of non-responders (38). Whilst the results of our study are largely confirmatory, the power conferred by the very large sample provides important validation and is a fundamental tool for individual or personalized risk prediction. As previously explored in our published descriptive study on the same pooled cohort (12), the wider availability, increased choice of DMTs and patient preferences also permit important lifestyle considerations to factor into treatment selection, including pregnancy planning, the management of side effects, work commitments, and travel (39, 40). A key limitation of this study was the lack of sufficient MRI data to include in the predictor analysis. Whilst the effects observed in the regression modeling are independent of confounding from the clinical and demographic included also in the multivariate models (i.e., age, sex, disease duration, time since diagnosis, baseline EDSS, and treatment calendar year/epoch), they do not control for other potential sources of confounding or heterogeneity between registries, including MRI lesion metrics.

## Data availability statement

The data analyzed in this study is subject to the following licenses/restrictions: Data availability is subject to the rules and governance of each participating registry. Requests to access these datasets should be directed to TSp, tim.spelman@ki.se.

## Author contributions

TSp: Conceptualization, Data curation, Formal analysis, Investigation, Methodology, Resources, Software, Supervision, Validation, Writing—original draft, Writing—review & editing. MM: Conceptualization, Writing—review & editing. HB: Conceptualization, Writing—review & editing. AV: Writing—review & editing. SV: Conceptualization, Writing—review & editing. MTr: Conceptualization, Writing—review & editing. PI: Writing—review & editing. DH: Conceptualization, Writing—review & editing. JD: Conceptualization, Writing—review & editing. FP: Writing—review & editing. RH: Conceptualization, Writing—review & editing. PD: Writing—review & editing. JL-S: Writing—review & editing. SS: Writing—review & editing. PL: Writing—review & editing. VS: Writing—review & editing. SO: Writing—review & editing. SE: Writing—review & editing. RAl: Writing—review & editing. MTe: Writing—review & editing. MG: Writing—review & editing. TK: Writing—review & editing. FG'M: Writing—review & editing. OS: Writing—review & editing. SK: Writing—review & editing. BY: Writing—review & editing. MS: Writing—review & editing. OG: Writing—review & editing. YB: Writing—review & editing. RK: Writing—review & editing. CO-G: Writing—review & editing. AA: Writing—review & editing. SH: Writing—review & editing. PM: Writing—review & editing. RAm: Writing—review & editing. KdG: Writing—review & editing. CM: Writing—review & editing. AS: Writing—review & editing. JP: Writing—review & editing. NJ: Writing—review & editing. JI: Writing—review & editing. LS: Writing—review & editing. AM: Writing—review & editing. LF: Writing—review & editing. FS: Writing—review & editing. AG: Funding acquisition, Project administration, Writing—review & editing. LP: Writing—review & editing. HJ: Writing—review & editing. PR: Writing—review & editing. TSe: Writing—review & editing. MP: Writing—review & editing. JC: Writing—review & editing. MK: Writing—review & editing. MS: Writing—review & editing. HM: Writing—review & editing. JH: Conceptualization, Funding acquisition, Project administration, Supervision, Writing—review & editing.

## References

[1] Fymat AL. Multiple sclerosis: II. Diagnosis and symptoms management. J Neurol Psychol Res. (2023) 4:1–21.

[2] Harding K Williams O Willis M Hrastelj J Rimmer A Joseph F. Clinical outcomes of escalation vs early intensive disease-modifying therapy in patients with multiple sclerosis. JAMA Neurol. (2019) 76:536–41. 10.1001/jamaneurol.2018.4905 30776055 PMC6515582

[3] Spelman T Magyari M Piehl F Svenningsson A Rasmussen PV Kant M. Treatment escalation vs immediate initiation of highly effective treatment for patients with relapsing-remitting multiple sclerosis: Data from 2 different national strategies. JAMA Neurol. (2021) 78:1197–204. 10.1001/jamaneurol.2021.2738 34398221 PMC8369379

[4] Lorscheider J Benkert P Lienert C Hänni P Derfuss T Kuhle J. Comparative analysis of dimethyl fumarate and fingolimod in relapsing–remitting multiple sclerosis. J Neurol. (2021) 268:941–9. 10.1007/s00415-020-10226-6 32974794 PMC7914245

[5] Papp V Buron MD Siersma V Rasmussen PV Illes Z Kant M. Real-world outcomes for a complete nationwide cohort of more than 3200 teriflunomide-treated multiple sclerosis patients in The Danish Multiple Sclerosis Registry. PLoS ONE. (2021) 16:e0250820. 10.1371/journal.pone.0250820 34003862 PMC8130956

[6] Setayeshgar S Kingwell E Zhu F Zhang T Carruthers R Marrie RA. Persistence and adherence to the new oral disease-modifying therapies for multiple sclerosis: a population-based study. Mult Scler Relat Disord. (2019) 27:364–9. 10.1016/j.msard.2018.11.004 30476872

[7] Evans C Marrie RA Zhu F Leung S Lu X Melesse DY. Adherence and persistence to drug therapies for multiple sclerosis: a population-based study. Mult Scler Relat Disord. (2016) 8:78–85. 10.1016/j.msard.2016.05.006 27456879

[8] Warrender-Sparkes M Spelman T Izquierdo G Trojano M Lugaresi A Grand'Maison F. The effect of oral immunomodulatory therapy on treatment uptake and persistence in multiple sclerosis. Multiple Sclerosis J. (2016) 22:520–532. 10.1177/1352458515594041 26199347

[9] Bigaut K Cohen M Durand-Dubief F Maillard E Planque E Zephir H. How to switch disease-modifying treatments in multiple sclerosis: guidelines from the French Multiple Sclerosis Society (SFSEP). Mult Scler Relat Disord. (2021) 103076. 10.1016/j.msard.2021.103076 34161898

[10] Patti F Chisari CG D'Amico E Annovazzi P Banfi P Bergamaschi R. Clinical and patient determinants of changing therapy in relapsing-remitting multiple sclerosis (SWITCH study). Mult Scler Relat Disord. (2020) 42:102124. 10.1016/j.msard.2020.102124 32353755

[11] Trojano M Tintore M Montalban X Hillert J Kalincik T Iaffaldano P. Treatment decisions in multiple sclerosis—insights from real-world observational studies. Nat Rev Neurol. (2017) 13:105–18. 10.1038/nrneurol.2016.188 28084327

[12] Hillert J Magyari M Soelberg Sørensen P Butzkueven H Van Der Welt A Vukusic S. Treatment switching and discontinuation over 20 years in the big multiple sclerosis data network. Front Neurol. (2021) 12:295. 10.3389/fneur.2021.647811 33815259 PMC8010264

[13] Stühler E Braune S Lionetto F Heer Y Jules E Westermann C. Framework for personalized prediction of treatment response in relapsing remitting multiple sclerosis. BMC Med Res Methodol. (2020) 20:1–15. 10.1186/s12874-020-0906-6 32028898 PMC7006411

[14] Rotstein D Montalban X. Reaching an evidence-based prognosis for personalized treatment of multiple sclerosis. Nat Rev Neurol. (2019) 15:287–300. 10.1038/s41582-019-0170-8 30940920

[15] Chitnis T Prat A. A roadmap to precision medicine for multiple sclerosis. Multiple Scler J. (2020) 26:522–32. 10.1177/1352458519881558 31965904

[16] Havas J Leray E Rollot F Casey R Michel L Lejeune F. Predictive medicine in multiple sclerosis: a systematic review. Mult Scler Relat Disord. (2020) 40:101928. 10.1016/j.msard.2020.101928 32004856

[17] Iaffaldano P Lucisano G Butzkueven H Hillert J Hyde R Koch-Henriksen N. Early treatment delays long-term disability accrual in RRMS: results from the BMSD network. Multiple Sclerosis J. (2021) 27:13524585211010128. 10.1177/13524585211010128 33900144

[18] Kalincik T Butzkueven H. The MSBase registry: informing clinical practice. Multiple Scler J. (2019) 25:1828–34. 10.1177/1352458519848965 31120376

[19] Hillert J Stawiarz L. The Swedish MS registry–clinical support tool and scientific resource. Acta Neurol Scand. (2015) 132:11–9. 10.1111/ane.12425 26046553 PMC4657484

[20] Magyari M Joensen H Laursen B Koch-Henriksen N. The Danish multiple sclerosis registry. Brain Behav. (2021) 11:e01921. 10.1002/brb3.1921 33128351 PMC7821574

[21] Trojano M Bergamaschi R Amato MP Comi G Ghezzi A Lepore V. The Italian multiple sclerosis register. Neurol Sci. (2019) 40:155–65. 10.1007/s10072-018-3610-0 30426289 PMC6329744

[22] Vukusic S Casey R Rollot F Brochet B Pelletier J Laplaud DA. Observatoire Français de la Sclérose en Plaques (OFSEP): a unique multimodal nationwide MS registry in France. Multiple Scler J. (2020) 26:118–22. 10.1177/1352458518815602 30541380

[23] Spelman T Horakova D Ozakbas S Alroughani R Onofrj M Kalincik T. Switching to natalizumab or fingolimod in multiple sclerosis: comparative effectiveness and effect of pre-switch disease activity. Multiple Scler Relat Disor. (2023) 70:104477. 10.1016/j.msard.2022.104477 36746088

[24] Gutierrez RG. Parametric frailty and shared frailty survival models. Stata J. (2002) 2:22–44. 10.1177/1536867X0200200102

[25] Meyniel C Spelman T Jokubaitis VG Trojano M Izquierdo G Grand'Maison F. Country, sex, EDSS change and therapy choice independently predict treatment discontinuation in multiple sclerosis and clinically isolated syndrome. PLoS ONE. (2012) 7:e38661. 10.1371/journal.pone.0038661 22768046 PMC3387159

[26] Kalincik T Manouchehrinia A Sobisek L Jokubaitis V Spelman T Horakova D. Towards personalized therapy for multiple sclerosis: prediction of individual treatment response. Brain. (2017) 140:2426–43. 10.1093/brain/awx185 29050389

[27] Ayrignac X Bigaut K Pelletier J de Seze J Demortiere S Collongues N. First line treatment failure: Predictive factors in a cohort of 863 relapsing remitting MS patients. Mult Scler Relat Disord. (2021) 48:102686. 10.1016/j.msard.2020.102686 33340929

[28] Sorensen PS Kopp TI Joensen H Olsson A Sellebjerg F Magyari M. Age and sex as determinants of treatment decisions in patients with relapsing-remitting MS. Mult Scler Relat Disord. (2021) 50:102813. 10.1016/j.msard.2021.102813 33578207

[29] Patti F Chisari CG Arena S Toscano S Finocchiaro C Fermo SL. Factors driving delayed time to multiple sclerosis diagnosis: results from a population-based study. Mult Scler Relat Disord. (2022) 57:103361. 10.1016/j.msard.2021.103361 35158432

[30] Ghezzi A. European and American guidelines for multiple sclerosis treatment. Neurol Ther. (2018) 7:189–94. 10.1007/s40120-018-0112-1 30315481 PMC6283786

[31] Ziemssen T Derfuss T de Stefano N Giovannoni G Palavra F Tomic D. Optimizing treatment success in multiple sclerosis. J Neurol. (2016) 263:1053–65. 10.1007/s00415-015-7986-y 26705122 PMC4893374

[32] Gross RH Corboy JR. Monitoring, switching, and stopping multiple sclerosis disease-modifying therapies. Continuum. (2019) 25:715–35. 10.1212/CON.0000000000000738 31162313

[33] Hauser SL Cree BA. Treatment of multiple sclerosis: a review. Am J Med. (2020) 133:1380–90. 10.1016/j.amjmed.2020.05.049 32682869 PMC7704606

[34] Wattjes MP Ciccarelli O Reich DS Banwell B de Stefano N Enzinger C. 2021 MAGNIMS–CMSC–NAIMS consensus recommendations on the use of MRI in patients with multiple sclerosis. Lancet Neurol. (2021) 20:653–70. 10.1016/S1474-4422(21)00095-8 34139157

[35] Cross A Riley C. Treatment of multiple sclerosis. Continuum. (2022) 28:1025–51. 10.1212/CON.0000000000001170 35938656

[36] Stankiewicz JM Weiner HL. An argument for broad use of high efficacy treatments in early multiple sclerosis. Neurol Neuroimmunol Neuroinflam. (2020) 7:636. 10.1212/NXI.0000000000000636 31757815 PMC6935832

[37] Cree BA Mares J Hartung HP. Current therapeutic landscape in multiple sclerosis: an evolving treatment paradigm. Curr Opin Neurol. (2019) 32:365–77. 10.1097/WCO.0000000000000700 30985372

[38] Sabathé C Casey R Vukusic S Leray E Mathey G De Sèze J. Improving the decision to switch from first-to second-line therapy in multiple sclerosis: a dynamic scoring system. Multiple Scler J. (2023) 29:236–47. 10.1177/13524585221139156 36515394

[39] Berger T Adamczyk-Sowa M Csépány T Fazekas F Fabjan TH Horáková D. Factors influencing daily treatment choices in multiple sclerosis: practice guidelines, biomarkers and burden of disease. Ther Adv Neurol Disord. (2020) 13:1756286420975223. 10.1177/1756286420975223 33335562 PMC7724259

[40] Toscano S Chisari CG Meli A Finocchiaro C Fermo SL Zappia M. Pregnancy planning and management for women with multiple sclerosis: what has changed over the last 15 years? An Italian single-center experience. Multiple Scler Relat Disor. (2023) 104526. 10.1016/j.msard.2023.104526 36689891

